# The Impact of COVID-19 Pandemic Lockdown on the Incidence of New-Onset Type 1 Diabetes and Ketoacidosis Among Saudi Children

**DOI:** 10.3389/fendo.2021.669302

**Published:** 2021-04-01

**Authors:** Aqeel Alaqeel, Fahad Aljuraibah, Mohammed Alsuhaibani, Mohammed Huneif, Abdulhameed Alsaheel, Mohammad Al Dubayee, Abdulaziz Alsaedi, Ayman Bakkar, Ahmed Alnahari, Areej Taha, Khulood Alharbi, Yousef Alanazi, Samia Almadhi, Reem Al Khalifah

**Affiliations:** ^1^ Department of Pediatrics, College of Medicine, Qassim University, Buraidah, Saudi Arabia; ^2^ Pediatric Department, King Abdullah Specialized Children’s Hospital, Riyadh, Saudi Arabia; ^3^ College of Medicine, King Saud bin Abdulaziz University for Health Sciences, Riyadh, Saudi Arabia; ^4^ King Abdullah International Medical Research Center (KAIMRC), Ministry of National Guard-Health Affairs (MNGHA), Riyadh, Saudi Arabia; ^5^ Department of Pediatrics, College of Medicine, Najran University, Najran, Saudi Arabia; ^6^ Pediatric Endocrine Department, Obesity, Endocrine and Metabolism Center, King Fahad Medical City, Riyadh, Saudi Arabia; ^7^ Al Hada Armed Forces Hospital, Pediatric Department, Pediatric Endocrine Division, Taif, Saudi Arabia; ^8^ Diabetes and Endocrinology Center, King Fahad Central Hospital in Jizan, Jizan, Saudi Arabia; ^9^ Pediatric Endocrinology Division, Pediatrics Department, College of Medicine, King Saud University, Riyadh, Saudi Arabia; ^10^ Department of Pediatrics, College of Medicine, Taibah University, Madinah, Saudi Arabia; ^11^ Department of Pediatrics, College of Medicine, Majmaah University, Majmaah, Saudi Arabia

**Keywords:** type 1 diabetes mellitus, DKA, Saudi Arabia (KSA), COVID-19 pandemic, lockdown

## Abstract

**Background:**

Overburdened healthcare systems during the coronavirus disease (COVID-19) pandemic led to suboptimal chronic disease management, including that of pediatric type 1 diabetes mellitus (T1DM). The pandemic also caused delayed detection of new-onset diabetes in children; this increased the risk and severity of diabetic ketoacidosis (DKA). We therefore investigated the frequency of new-onset pediatric T1DM and DKA in Saudi Arabia during the COVID-19 pandemic and compared it to the same period in 2019.

**Methods:**

We conducted a multicenter retrospective cohort study, including patients aged 1–14 years admitted with new-onset T1DM or DKA during the COVID-19 pandemic (March–June 2020) and the same period in 2019. We assessed factors including age, sex, anthropometric measures, nationality, duration of diabetes, diabetes management, HbA1c levels, glycemic control, cause of admission, blood gas levels, etiology of DKA, DKA complications, length of hospital stay, and COVID-19 test status.

**Result:**

During the lockdown, 106 children, compared with 154 in 2019, were admitted to 6 pediatric diabetes centers. Among the admissions, DKA was higher in 2020 than in 2019 (83% vs. 73%; *P*=0.05; risk ratio=1.15; 95% confidence interval, 1.04–1.26), after adjusting for age and sex. DKA frequency among new-onset T1DM and HbA1c levels at diagnosis were higher in 2020 than in 2019 (26% vs. 13.4% [*P*=<0.001] and 12.1 ± 0.2 vs. 10.8 ± 0.25 [*P*<0.001], respectively). Females and older patients had a higher risk of DKA.

**Conclusion:**

The lockdown implemented in Saudi Arabia has significantly impacted children with T1DM and led to an increased DKA frequency, including children with new-onset T1DM, potentially owing to delayed presentation.

## Introduction

The prevalence of type 1 diabetes mellitus (T1DM) in Saudi Arabia is considered to be among the highest globally (1). One of the major and life-threatening complications of T1DM is diabetic ketoacidosis (DKA) as it may potentially lead to significantly higher rates of morbidity and mortality, along with impacting families and increasing the burden on healthcare systems (2, 3). Unfortunately, the incidence of DKA in Saudi Arabia remains as high as 40–70% among patients with new-onset T1DM, which is considerably high when compared with that in other countries (4, 5). This may have worsened during the coronavirus disease (COVID-19) pandemic when access to healthcare was insufficient.

Globally, the implemented lockdowns during the COVID-19 pandemic impacted access to healthcare for patients with chronic disorders including children with T1DM (6–8). Reports from the UK, Germany, the USA, Italy, China, and Spain documented an increase in the frequency and severity of DKA among children with T1DM (9–15). The government of Saudi Arabia had implemented early measures to reduce virus transmission prior to COVID-19 being declared a pandemic. However, in March 2020, after the World Health Organization declared the COVID-19 outbreak a pandemic, and owing to an increase in infected cases, Saudi Arabia imposed further preventive measures including a nationwide lockdown, banning international and national travel, closing of schools, and suspension of elective procedures and non-essential visits to hospitals (16, 17). The lockdown was lifted by end of June 2020, and ever since, other restrictions have been gradually minimized, as of January 2021 (18, 19).

The lockdown, local travel restrictions, virtual clinics instead of routine visits, suspension of non-essential hospital visits, and increased fear of acquiring COVID-19 infections from hospitals are the potential factors for delayed presentation of diagnosed and undiagnosed T1DM. In this study, we aimed to compare the frequency and severity of DKA in children with T1DM from six large pediatric diabetes centers in Saudi Arabia between March 1 and June 30 in 2019 and 2020 to examine the effect of the pandemic.

## Materials and Methods

### Population and Sampling

We conducted a retrospective multicenter cohort study that included six pediatric diabetes centers in major cities of Saudi Arabia. We included patients aged 1–14 years, who were admitted with new-onset T1DM or DKA between March 1, 2020, and June 30, 2020, and compared their data to that of patients admitted in the same period of 2019. We excluded patients with pre-existing T1DM who were admitted for other reasons such as hypoglycemia, hyperglycemia without ketoacidosis, or surgical reasons. T1DM patients were identified either through electronic medical records using ICD-9 and ICD-10 codes for diabetes and insulin use during emergency room visits or inpatient admissions while some centers used their database registry to identify eligible patients. The data were collected using an electronic Google form.

The study was approved by the Institutional Review Board or the Independent Ethics Committee of each site prior to the initiation of the study. All aspects of the study were conducted in accordance with the International Council on Harmonization Good Clinical Practice principle.

### Measures

We collected data on age, sex, anthropometric measures, nationality, duration of diabetes, diabetes management, latest HbA1c reports, average of the last 3 HbA1c reports, cause of admission, blood gas levels, etiology of DKA, DKA complications, length of stay in intensive care, and length of hospital stay. The diagnosis of diabetes was based on the American Diabetes Association criteria (20). T1DM was diagnosed if patients had at least one anti-pancreatic antibody positive, without features suggestive of type 2 diabetes and other forms of diabetes. The diagnosis of DKA was defined as a pH level <7.3 and/or bicarbonate level <15 mmol/L, along with ketonemia or ketonuria in the presence of hyperglycemia (blood glucose >11 mmol/L [≈200 mg/dL]) and severe DKA as pH <7.1 and/or serum bicarbonate <5 mmol/L (3).

### Outcomes

The primary outcome was the frequencies of new onset T1DM and DKA during the period of March to June of 2019 and 2020. The secondary outcomes included the percentage of children admitted with severe DKA, parentage of DKA among new onset T1DM. Severe DKA was defined as a pH level less than 7.1 and/or bicarbonate level less than 5 mmol/L.

### Statistical Analysis

We presented dichotomous data as frequencies (n) and proportions (%) and continuous data as mean ± standard error of the mean (SEM) for normally distributed data. We compared the frequencies of T1DM, DKA, and severe DKA observed during the aforementioned periods using the χ^2^ test, and the data were adjusted using a generalized linear model for age and sex. We reported the effect estimate with risk ratio (RR) with 95% confidence intervals. Data analysis was performed using Stata/SE 16.0 for Mac.

## Results

A total of 260 children with T1DM were admitted to six pediatric diabetes centers in March–June 2019 and 2020 periods, of whom 154 children were admitted during 2019, and 106 children were admitted, in 2020. Overall, 200 (76.9%) children were admitted due to DKA, 112 of 154 patients (72.7%) in 2019 and 88 of 106 patients (83%) in 2020. Whereas overall admissions with new-onset T1DM was 98 (37.7%) in 2019 and 2020 (37% vs 38.7% respectively). The proportion of DKA among new-onset T1DM was significantly higher in 2020 (26%) than in 2019 (13.4%) [*P*=<0.001] (**Table 1**).

**Table 1:** DKA and severe DKA from March to June 2020, during the COVID-19 pandemic, compared with the same period in 2019.

		Overall N = 260	2019 N = 154	2020 N = 106	*P*-value
**Overall**	**Known T1DM**	162(62.3)	97(62.9)	65(61.3)	0.8
	**New-onset T1DM**	98 (37.7)	57 (37.0)	41 (38.7)	0.70
DKA*	**Overall**	200 (76.9)	112 (72.7)	88 (83.02)	0.05
	**New-onset**	35 (36.5)	15 (13.4)	23 (26)	<0.001
	**Known T1DM**	165(82.5)	97(86.6)	65(73.8)	0.89
Severe DKA*	**Overall**	47 (18.1)	24 (15.6)	23 (21.7)	0.20
	**New-onset**	11(23.4)	4(16.6)	7(30.4)	0.13
	**Known T1DM**	36 (76.6)	20(83.3)	16(69.5)	0.60

The mean age of the children was 9.8 ± 0.2 years, 120 (46.2%) were male, and 240 (95.4%) were Saudi Arabian (**Table 2**). Although the mean body-mass index (BMI) and BMI standard deviation score (SDS) were within the normal range, the study population showed higher BMI and BMI SDS in 2020, with a mean BMI z-score difference of −0.47 ± 0.28. Among 162 (62.3%) children with known T1DM, the mean diabetes duration was 4.8 ± 0.8 years. The mean HbA1c level was higher in 2020 than in 2019, with a mean difference of −0.7 ± 0.3% (*P*=0.13). HbA1c levels at diagnosis were significantly higher in 2020 than in 2019 (12.1 ± 0.2 vs. 10.8 ± 0.2.5) [*P*<0.001]). In children with established T1DM, most used multiple daily injections (91.9%), followed by insulin pump (6.5%), and two daily injections (4.9%). One child was reported to have developed cerebral edema in 2020, while 15 had developed acute kidney injury in 2019, and one in 2020 and no mortality secondary to DKA in both years.

**Table 2:** Baseline characteristics.

	Overall N = 260	2019 N = 154	2020 N = 106	*P*-value
**Age, years**	9.8 (0.2)	9.7 (0.24)	10.0 (0.3)	0.2
Sex, male*	120 (46.2)	69 (44.8)	51 (48.1)	0.6
**Diabetes duration**	4.8 (0.8)	5.1 (1.3)	4.4 (0.3)	0.6
**BMI, z-score**	−0.66 (0.1)	−0.85 (0.17)	−0.39 (0.22)	0.05
**HbA1c, %**	11.6 (0.1)	11.3 (0.2)	12.1 (0.2)	<0.001
**HbA1c% among new-onset**	11.5 (2.2)	10.87 (2.5)	12.14 (2.1)	<0.001
**HbA1c% among known T1DM**	11.70 (1.7)	11.57 (1.8)	12.0 (1.6)	0.13
**Average of last 3 HbA1c, %**	10.9 (0.2)	10.9 (0.2)	10.9 (0.2)	0.5
**PICU length of stay, days**	0.55 (0.04)	0.5 (0.1)	0.6 (0.1)	0.08
**Hospital length of stay, days**	2.88 (0.1)	2.9 (0.1)	2.9 (0.2)	0.5

Values are mean ± SEM unless indicated.

*N (%).

The frequency of admission for new-onset T1DM was similar in both years. In contrast, admission due to DKA incidence was higher among newly diagnosed T1DM in 2020 (unadjusted RR=2.1; 95% CI, 1.3, 3.6) than in 2019, and (RR= 1.15; 95CI 1.04, 1.26) after adjusting for age and sex (**Table 3**). Females and older children had a higher risk of developing DKA and severe DKA. The frequency of severe DKA was similar in both years, after adjusting for age and sex. In 2020, two children who were diagnosed with severe DKA tested positive for COVID-19.

**Table 3:** Adjusted risk ratio of DKA and severe DKA from March to June 2020, during the COVID-19 pandemic, compared with the same period in 2019.

	DKA				Severe DKA			
	Risk ratio	95% CI		*P*-value	Risk ratio	95% CI		*P*-value
Visit period, 2020^a^	1.15	1.04	1.26	0.01	1.37	0.83	2.25	0.22
Age, years^b^	1.05	1.03	1.07	0.00	1.15	1.04	1.27	0.01
Sex, male^c^	0.76	0.68	0.85	0.00	0.56	0.32	0.97	0.04
**Constant**	0.00	0.00	0.00	0.01	0.00	0.00	0.00	0.22

a Adjusted for current age and sex.

b Adjusted for visit period and sex.

c Adjusted for current age and visit period.

Risk factors leading to DKA among established cases include insulin omission (73%), infection (16%), insulin pump failure (1.2%), fasting (0.6%), improper insulin injection technique (0.6%), and other causes (0.6%).

## Discussion

To the best of our knowledge, this is the first multicenter study conducted in Saudi Arabia and the Middle East that compared the frequency of DKA during the COVID-19 pandemic to the same period in the previous year. Our study found a remarkable increase of 11% in DKA frequency among children with T1DM during the lockdown period. However, after adjusting for age and sex, there was no difference observed in DKA severity. Interestingly, despite a noticeable decrease in the frequency of new-onset T1DM in children, a significant increase was found in those children presenting with DKA, compared with those in 2019. A German study found increased incidence and severity of DKA during the pandemic on March 13, 2020, through May 13 compared to the same period of 2019 and 2018; however, the incidence of new-onset T1DM diagnosis remained the same (11). On the other hand, another study conducted in Italy between February 20 and April 14 in 2019 and 2020 found no difference in DKA incidence and new-onset T1DM diagnosis; however, an increase in DKA severity was observed (10). A survey from 88 diabetes centers in the UK between March 1, 2020, and June 30, 2020, showed an increase in the incidence and severity of DKA at diagnosis, and about 20% of the centers have attributed this to delayed presentation, while the incidence of new-onset T1DM diagnosis remained the same (12).

Another interesting finding in our data was higher HbA1c during the pandemic among new-onset T1DM with or without DKA, which could be linked to delayed presentation. This finding has not been reported in previous studies, but UK multicenter study reported HbA1c in the pandemic with no comparison (14).

The possible causes of delayed presentation and increased DKA are likely to be similar to those reported in other countries, such as the focus of frontline healthcare workers on COVID-19-related clinical manifestations. In addition, parents avoided seeking medical care when children had milder symptoms with the concern of contracting COVID-19 at the hospital and owing to difficulties in accessing healthcare services due to the lockdown (6, 9).

Despite the relatively short period of lockdown, unfortunately, the BMI of children was found to be significantly higher in 2020 than in 2019. This finding was not reported in other T1DM cohorts investigated during the same period. However, studies on healthy children reported lower physical activity and increased screen time, which are considered major factors leading to childhood obesity (21).

Based on our data and those of other groups, we emphasize the recommendation of Elbarbary et al. that dissemination of awareness regarding T1DM symptoms in the public should be continued during a pandemic (9). Furthermore, telemedicine technology has been an effective tool during this pandemic to deliver care for known and new-onset diabetic patients. Thus, in such scenarios in the future, technology and telemedicine should be used to fill the gaps when the healthcare system is overburdened (6–9, 22).

There is a paucity of data regarding the severity of COVID-19 among children with T1DM (13). While adults with diabetes who contracted COVID-19 showed a severe disease course and poor outcome, studies in children with diabetes have demonstrated a milder course and better outcome (10, 22–24). Beliard et al. showed that DKA presentation among children with T1DM who were diagnosed with COVID-19 was not significantly different from those who were not (15). In the present study, COVID-19 cases among admitted patients were rare (2/106 patients). However, both patients had severe DKA with no complications related to COVID-19. Our findings are consistent with studies that showed a low prevalence of COVID-19 among children with diabetes (25).

Our study, however, has some limitations, the major one being that not all pediatric diabetes centers in Saudi Arabia were included; hence, we were unable to present region-adjusted incidence.

We have learned from our data as well as the previous studies that the pandemic has significantly impacted children with T1DM. The pandemic has caused a delay in the diagnosis of diabetes, which markedly increased DKA frequency and severity. This data emphasizes that healthcare access to diagnosed and undiagnosed children with diabetes should continue in the standard level of care during pandemics, in addition to the need for continuing awareness about T1DM to the public and among frontline healthcare practitioners.

## Data Availability Statement

The original contributions presented in the study are included in the article/supplementary material. Further inquiries can be directed to the corresponding author.

## Author Contributions

AAla, MA, RA, FA, and MD designed the research study. MH, AAln, AAls, AAls, AB, KA, YA, SA, and AT collected the data. AAla and RA analyzed the data. AAla, MA, RA, and FA wrote the first draft. All authors participated in the final draft. AAla and RA take final responsibility for this article. All authors contributed to the article and approved the submitted version.

## Funding

The researchers would like to thank the Deanship of Scientific Research, Qassim University, for funding the publication of this project.

## Conflict of Interest

The authors declare that the research was conducted in the absence of any commercial or financial relationships that could be construed as a potential conflict of interest.
